# ProLego: tool for extracting and visualizing topological modules in protein structures

**DOI:** 10.1186/s12859-018-2171-9

**Published:** 2018-05-04

**Authors:** Taushif Khan, Shailesh Kumar Panday, Indira Ghosh

**Affiliations:** School of Computational and Integrative Sciences, Jawaharlal Nehru University, New Delhi, 110067 India

**Keywords:** Protein topology, Topology comparisons., Protein Graph., Visualization., Server application.

## Abstract

**Background:**

In protein design, correct use of topology is among the initial and most critical feature. Meticulous selection of backbone topology aids in drastically reducing the structure search space. With ProLego, we present a server application to explore the component aspect of protein structures and provide an intuitive and efficient way to scan the protein topology space.

**Result:**

We have implemented in-house developed “topological representation” in an automated-pipeline to extract protein topology from given protein structure. Using the topology string, ProLego, compares topology against a non-redundant extensive topology database (ProLegoDB) as well as extracts constituent topological modules. The platform offers interactive topology visualization graphs.

**Conclusion:**

ProLego, provides an alternative but comprehensive way to scan and visualize protein topology along with an extensive database of protein topology. ProLego can be found at http://www.proteinlego.com

**Electronic supplementary material:**

The online version of this article (10.1186/s12859-018-2171-9) contains supplementary material, which is available to authorized users.

## Background

Understanding of protein fold universe remains one of the major goal in post genomic era. Numerous attempts in exploring the nature of protein structure space led to classification schemas like SCOP [1], CATH [2], ECOD [3] PCBOST [4] that investigate protein’s structural, functional and evolutionary features. Topology based approach has been recently exploited to examine the structure space of proteins and provide insights into fold designing and evolution [4–7]. Topology has been used extensively to address the nature of folding profile by both experimental and computational approaches [8]. Rockline et al. [9], recently reported improvements in protein designing with extensive use of high-throughput topology scanning in case of 4 mini-proteins. The use of topology in the context of protein designing, folding and stability studies has been widely used.

Structural modularity is crucial in conferring functional and structural diversity of proteins [6, 10]. This concept can be explained using analogy of structural modules as “Lego” blocks that can be reused to build proteins with tailored functionality. Although the analogy with “Lego” blocks might oversimplify the complex nature but can depict well the current understanding of protein structure space [11]. Protein topology has been studied using several graph-based techniques to understand domain arrangement [12, 13], protein folding pathways [8], analysis of different biochemical activities and structural comparison [14]. With the emergence of computer graphics, protein topology representation evolved from manual drawing [15] to scalable graphics representation [16, 17]. However, only handful of methods are available that provide automatic generation of the protein topology diagram (Additional file 1 section 1.1 and Table S1). The most recent addition in the list is Protein Topology Graph Library (PTGL) [17]. PTGL is a continuously developing topology library with the aim to provide protein folding graphs [18, 19]. However, the issue of module identification and visualisation could be addressed in much efficient way as proposed by protein lego server, as reported here.

With ProLego, we propose a platform that can be used to analyse protein topology and its modular architecture. ProLego, along with generating improved topology cartoon diagrams, provide tools for searching proteins with similar topology and extracting constituent structural modules. With the implementation of protein “topology string” on non-redundant protein chains, we propose a protein topology database (ProLegoDB) focusing on the composition and organisation of secondary structures.

## Implementation

ProLego is a “pythonic” solution to the topology generation with the help of D3.js (a JavaScript library) for visualization. Briefly, in the background, user provided protein chain examined for secondary structure (SS) contacts and relative orientation. The SS-contact definition is considered based on the presence of corresponding residual contacts (as in [14, 20]). From protein’s atomic coordinate, an adjacency matrix of SS-contact has been generated, from which 1D “topology string” has been built. The “topology string” encompasses the composition, contact and relative arrangement of SS (see Additional file 1 section S1.2).

The present study implements a pipeline that uses above mentioned (a) “Topology String” (or Contact String) to define relative position and orientation of secondary structure elements (SSE; DSSP definition [21]), (b) a database “ProLegoDB” of pre-calculated topology information of representative proteins [10] and (c) provides a topology visualization platform. “Topology String” translates protein topology in an intuitive character string, which has been further used in searching and storing of topologies. The architecture of the server is discussed in Additional file 1: Figure S1.

## Results

**Fig. 1:**
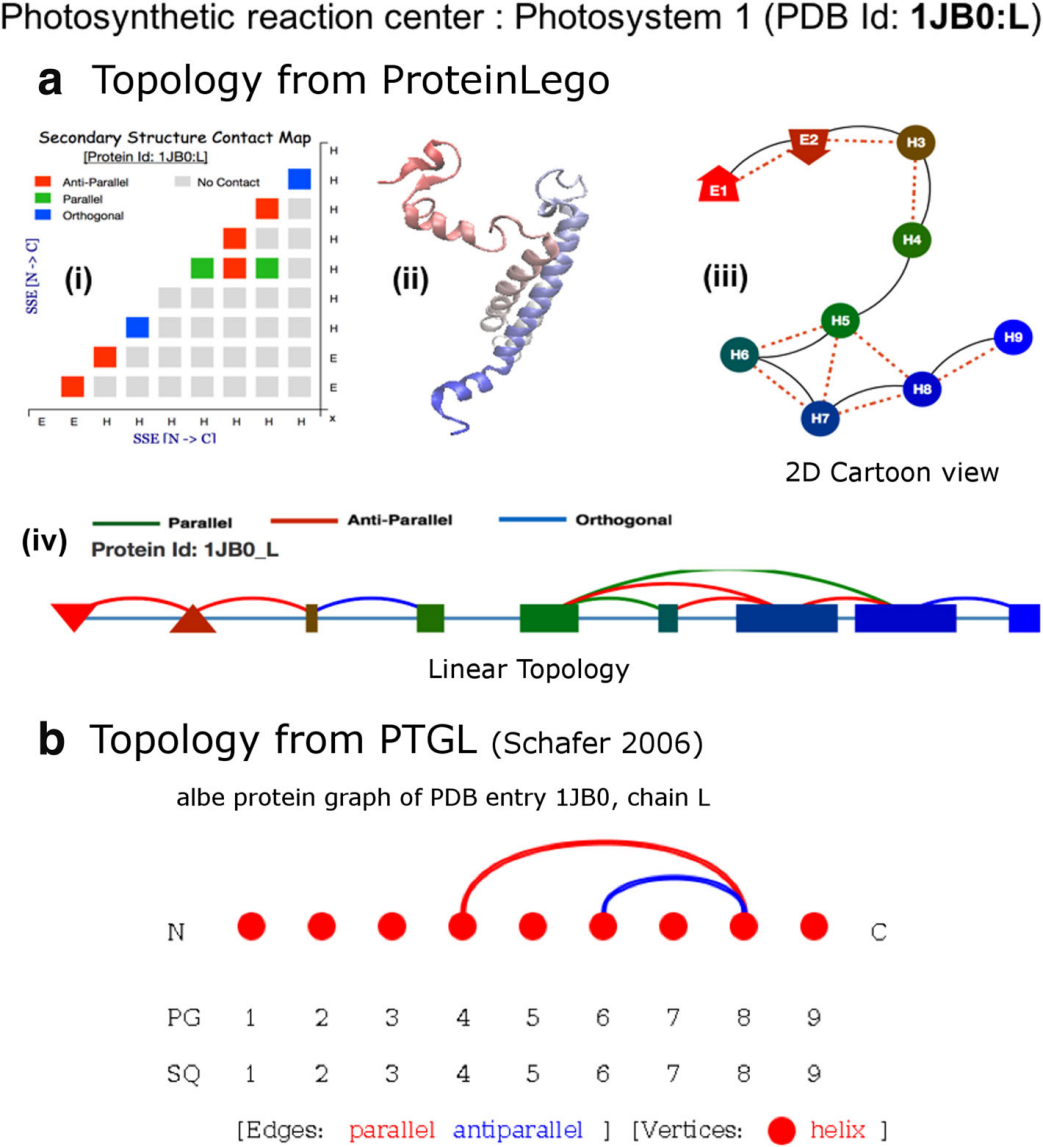
Comparing topology visualization using ProLego (**a**) and PTGL (**b**) for the case of photosynthetic reaction centre (Photosystem1 (PDB Id: 1JB0; chain: L)). The chain an anit-parallel beta sheet at the N-terminal followed by seven alpha-helices. Fig **a**.ii, shows a cartoon representation of protein chain using VMD. In linear topology (**a**.iv) strands are represented as triangles (with relative orientation as up/down triangle) and helices are represented as rectangle. The length of helical rectangles scaled as per number of residues in corresponding helix. The protein chain is represented as red to green to blue as passes from N to C terminal. The linear lines, connecting secondary structure (SS) blocks shows chain connectivity, whereas the arc lines represent spatial connectivity and type of SS contact (colour coded as labelled in Additional file 1: Table S4). The secondary structure contact map (**a**.i), shows all spatial contact between pairs of SS. A 3D carton representation (VMD generated **a**.ii) and 2D topology cartoon (**a**.iii) plot is generated from ProLego. The 2D ProLego cartoon shows contact between two SS blocks by red dotted lines and chain connectivity by black continuous line. Figure **b**, shows the topology representation of same protein generated using protein topology graph library (http://ptgl.uni-frankfurt.de/api/index.php/pg/1jb0/L/albe/json), the alpha-beta graph. The graph represents SSEs from N to C terminal in left to right fashion. Helices are represented as circles and stands as rectangles. PTGL considers, 3_10_ helices also in total helix, hence the addition of 1st and 7th helix, giving total number of helix to 9 instead of 7 alpha-helix as per ProLego in this protein. PTGL misses the N-terminal sheet, which is represented as up-down triangle (for anti-parallel orientation) in case of ProLego

ProLego leverages the component approach of protein topology space to extract inherent modules, similar topology and assigns topology frequency class (Preferred, Non-preferred). Representing protein topology as a graph of secondary structure, ProLego provides visualization focusing on different representation (Fig. 1a). ProLegoDB is an extensive database of protein topology generated by analysis of representative datasets (Additional file 1 section 1.7). The proposed web platform provides an intuitive approach to explore the protein structure topology space. The backend use of string-based (“topology string”) search method makes the process efficient and intuitive. In the following section, some of the key finding in nature of topology space by analysis of different representative non-redundant datasets have been discussed. Some of the salient features of the server application has been presented along with a comparative study with current state-of-the-arts topology servers.

### Distribution of proteins in topology space

Using secondary structures (SS) as building blocks of protein structure, we have defined topology as the arrangement, spatial contacts and organisation of SS in a protein chain. Applying this simple but efficient definition, we have scanned representative protein structure databases and extracted underlying topological space. The representative data sets have been curated for sequence redundancy with state-of-the-art methods to mitigate the effect of structural bias in current protein structure space. A brief description of dataset statistics can be read from Table 1 and from Additional file 1 section 1.7.

**Table 1:** Description of ProLegoDB

Structure Class	Topologies^a^	Proteins^b^	Domains^c^
A	3315	6064	2134
B	2485	3955	1520
Mix AB	1401	48,167	10,754
Total	7201	58,186	14,408

The topology database, ProLegoDB, describes protein topology space. Representative datasets of non-redundant protein chains and domain has been constructed as described in (S1.3). Above table summarises the database with different structure class (A: all-alpha, B: all-beta and mix AB: Alpha-Beta). Number of ^a^statistically significant topology group for each structure classes has been shown with table heading of “Topologies”. Number of proteins in the database for each structure class has been reported in the next columns. ^b^Protein chains are considered from extracted non- redundant datasets of PDB, whereas ^c^Domains are protein entry from curated domain databases of CATH (3.5) and Astral (SCOP v1.75). The maximum pairwise sequence identity between chains are < 40%

**Fig. 2:**
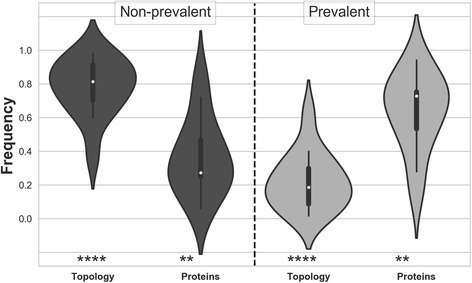
Distribution of topology and protein in groups of “Non-Prevalent” (left to dashed line) and “Prevalent” (right to dashed lines) has been shown as violin plots. This plot is generated for the statistically significant topologies (*P*-value < 0.001; Additional file 1: Table S3), from represented dataset of PDB (58,186 protein chains). Description of dataset has been provided in the text and supplementary. The shape of violin plot describes the kernel density estimation of the distribution of data in different topologies and proteins. A summary of statistics can be drawn from the inner boxplot. The white dot represents the median, thick bar shows the interquartile range and thin line describes the 95% confidence interval. A clear distinction can be drawn on the nature of distribution of proteins as well as topologies in “Prevalent” and “Non-prevalent” groups. A comparison of distribution with non-parametric Wilcoxon rank-sum test has been performed and *P*-values are indicated as ‘*’ (‘****’: *P*-val < 0.001 and ‘**’: *P*-val < 0.01) in the bottom

Distribution of proteins in different topologies have been examined and statistically significant topologies are identified (*p*-value < 0.001). The significance is further examined with restricting false discovery rate to less than 0.1%, using p-value correction method [22] (Additional file 1: Table S5). Figure 2, describes the distribution of proteins in statistically significant topologies. We have compared the topology and protein space in “Prevalent” (P) and “Non-prevalent” (NP) classes. For each case, the density of distribution is represented by the width of the violin plot and the spread of the inter-quartile region describes the variation. Comparing the density distribution, a clear distinction in distributions of “P” and “NP” can be observed. For each case, maximum density of the data can be found around their respective mean and interquartile regions, whose values varies for topology and proteins in both cases. Examining the distributions, it can be observed that the topologies in “P” are only ~ 20% of the total topology space, whereas it caters to ~ 70% of total proteins, which is reverse in the case of “NP”. This characteristic of distribution for topology is quite evident, however proteins have subtle higher variance, distributed around mean of ~ 60% for “P” and ~ 40% for “NP”. Similar analysis has been performed for different datasets and among structural classes (Additional file 1: Figure S5). Among all studied cases, we have observed the consistent distribution of topology space, with tolerable variance in protein distribution across structure classes.

Using the topology string, it is possible to draw a distribution and study the variation in topology in protein structure space. The consistent observation of 80/20 rule in topology space is perceptible as shown by Fig. 1 and Additional file 1: Figure S5. This can be drawn in parallel to “Pareto-distribution” that is eminent in across fields of natural sciences and economics [23, 24]. The variance in protein space in “Prevalent” and “Non-prevalent” groups are majorly influenced by the nature of “structure class”. However, the emergent pattern of “small fraction of topology mostly populating structure space” can be drawn.

### Topology visualization

ProLego draws protein topology diagrams using “Topology String”. The pipeline extracts nodes (SSEs) and edges (sequential and spatial contact) using developed topology visualizer and renders in 2D and 1D SVG plots (Additional file 1 Section 1.6 and 1.7). Figure 1a, shows the ProLego result for photosynthetic reaction centre protein (PDB id: 1JB0, chain L). The protein chain has N-terminal β-hairpin, followed by seven α-helices. As shown by the Fig. 1a, ProLego generates (i) secondary structure contact map, (ii) 2D-cartoon view and (iii) linear topology graphs, representing different ways to examine protein topology. The secondary structure contact map illustrates the presence of contact and their relative orientation with different colour codes. Similar colour codes have been used in the linear topology which shows secondary structures from N to C terminal with strands as triangles (up/down relative to orientation) and rectangle blocks as alpha helices. Spatial contacts between SS have been shown as arcs. The cartoon view, illustrates the protein topology graph where solid lines show the sequential SSE contact whereas, the dashed red line shows the presence of tertiary contact between corresponding secondary structures.

### Extracting protein topology modules

This protocol extracts sub-structures or modules from a protein by analysing topology string. Fixing a window of one SSE from N to C-terminal, all observable protein topology in a chain has been listed (Additional file 1: Figure S2, Section 1.3). For a protein with “n” SSE (*n* > 3), the search extracts “n-1” SSE-topology modules, following the SSE combination stepwise from N to C-terminal. The topology database, ProLegoDB, are then used to map the protein chains and domains with each resultant topology modules. A working example of extracted topological modules for photoreaction centre protein (1JB0:L) has been discussed in Additional file 1: Table S3.

### Topology database

ProLegoDB is an extensive database for protein topology. The database is the collection of unique topologies extracted from non-redundant protein sets, generated from PDB (using PISCES-server [25]) and curated domain databases (Additional file 1 Section 1.7). This database has 58,186 protein chains and 14,408 protein domains topology analysed and grouped into 7201 statistically significant topology groups (Table 1). As the topologies are defined as per their secondary structure construct, its relatively easy to divide the whole space into all-alpha (A), all-beta (B) and alpha-beta (AB), structure classes. Each topology has been reported with observed occurrence frequency and statistical significance score (Additional file 1: Table S5).

A search in ProLegoDB can be performed from three levels i.e. Topology, Protein and Domains. Using “Search by Topology”, user can provide queries as per SS composition or advanced query of filtering with numbers of helix, strands as well as statistical significance. The query result lists all possible topologies with requested SS-composition along with their significant scores. Each row of the result has the corresponding link describing topology.

### Comparison of ProLego with PTGL

Among current state-of-the-art protein graph generation servers (Additional file 1: Table S1), PTGL is the most recent [17]. This is a subsequent upgrade and development over protein topology graph library [18, 19]. PTGL’s integration of graph modelling language (GML) for visualization is one of the first kind to apply in protein graphs (Fig. 1b). The most recent addition include ligand information in protein secondary structure contact and decomposes protein chain into alpha, beta and alpha-beta and receptor–ligand graphs [17]. The approach is shown to be used for searching sub-graphs, which is a crucial aspect of protein graph analysis, as also reported by Pro-Origami [16] and Tableaux [26].

Both PTGL and ProLego, address the topological graph from secondary structures. A comparative study on type of topology visualisation for PTGL and ProLego has been shown in Fig. 1. With ProLego, we illustrate the usability of string based topological representation. ProLego, provides more detailed and modular view to protein topology landscape. Our primary focus is to describe the variation in protein topology space, hence have not considered the ligand interactions. However, in the context of protein topology, ProLego provides topological frequencies (as P/NP) and statistical significance for all reported topologies. The extensive topology database, with different search modules, is advantageous to tailor search for topology. Identification of topological modules remains one of the most significant development in ProLego as compare to other topology databases.

### Application in protein designing

In protein designing, managing and filtering designed templates is one of the major challenges. In recent development in the field, Rocklin et al. [8], has reported successful designing of stable topologies in case of mini-proteins. In different rounds of optimisation, authors have generated *de-novo* decoys which provides an ideal synthetic dataset for investigating the occurrence of ProLego topology. Detail of experimental setup and dataset used has been discussed in Additional file 1 section 1.5. Investigating topologies in four mini proteins with secondary structure ααα, αββα, βαββ and ββαββ, we have observed different frequencies of “P” and “NP” topologies. For example, in simple three α topologies, ~ 90% of stable designs have prevalent topology. In case of βαββ and ββαββ, although number of examined topology increased, the presence of “stable” designs in prevalent topology classes remains significantly higher (Additional file 1: Table S7).

## Conclusion

With ProLego, we aim to provide an alternative approach to study protein structure topology. ProLego is inspired by modular architecture in protein topology space, which can be easily studied by the proposed “Topology String”. The component approach is found to be efficiently scanning the structure space and explore the nature of topology space. To understand the secondary structure based architecture in proteins, ProLego have compiled an extensive topology database analyzing different sets of non-redundant representative protein datasets. The server application provides an easy access to the database as well as enables users to investigate their protein of interest. With the integration of state-of-the-art framework and libraries, improved topology visualization approaches have been implemented and compared with other open source topology servers. Exclusively, ProLego-Server can be used for identifying constituent topological modules in proteins of interest, which could be used as “lego-blocks” in protein designing.

## Additional file

Additional file 1: a. File name: Supplimentary_material.pdf. b. Title of Data: Supplementary Information. c. Description of data: Supporting information for different experiments quoted in the main text. (PDF 2692 kb)

## Abbreviations

NP: Non-Prevalent topology
P: Prevalent topology
PDB: Protein Data Bank
SSE: Secondary structure elements
