# Supplementary material for: ProLego: tool for extracting and visualizing topological modules in protein structures
Source: BMC Bioinformatics. 2018 May 4;19:167. doi: 10.1186/s12859-018-2171-9 (PMC5935970; doi:10.1186/s12859-018-2171-9)
Supplement: Supplementary file 1 — a. File name: Supplimentary_material.pdf. b. Title of Data: Supplementary Information. c. Description of data: Supporting information for different experiments quoted in the main text. (PDF 2692 kb) [file 12859_2018_2171_MOESM1_ESM.pdf]

ProLego: tool for extracting and visualizing topological modules in protein structures

Supplementary Information

Taushif Khan, Shailesh Kumar Panday, Indira Ghosh

1.1 Approaches to analyze protein topology

Over the years, methods like PROMOTIF [1], TopDraw [2], TOPs+ [3], Pro-Origami [4]) and PTGL [5] have implemented different algorithms to generate protein topology graphs from protein 3D atomic structures. With the objective of understand and simplify the atomic structure space, topological approach has been used in different scales. However, only handful of methods are available that provide automatic generation of the protein topology diagram [4,5] (Table S1).

Table S1 : Building protein topology cartoon diagram.

Sl.	Method	General working principle	Reference	Remark/Availability
1	HERA (1990)	Based on H-bonds inspired from works of Jane Richardson (*1*)	Hutchinson and Thronton	Algorithm, no public web server
2	PRO-MOTIF (1996)	Provides details of the location of structural motifs in PDB. Used to compare protein topologies	Hutchinson *et.al*[1]	Stand-alone code available on request to the authors.
3	TOPs (2000)	Protein topology by analyzing relative position and orientation of secondary structures.	Westhead *et.al* (*[6]*)	http://balabio.dcs.gla.ac.uk/tops/index.html (Old version no update)
4	TopDraw (2003)	Manual drawing sheet for generating topology cartoon from TOPs definition. TopDraw requires Tcl/Tk version 8.0 or better.	Bond *et.al*[2]	Stand alone available from http://www.ccp4.ac.uk/html/topdraw.html
5	PDBSum (2005)	Uses HERA to represent protein topology in exploded fashion	Laskowski *et.al.*	http://www.ebi.ac.uk/pdbsum/
6	TOPs+ (2008)	Uses TOPs algorithm and includes feature like string notion.	Veeramali M and Gilbert D [3]	http://balabio.dcs.gla.ac.uk/mallika/WebTOPS/
7	PTGL (2010)	Generates protein topology graph based on interaction among secondary structures. Uses protocol described in by Prof. Ina Koch group (*10*, *11*)	May *et.al.*[5]	http://ptgl.uni-frankfurt.de/
8	Pro-Origami (2011-14)	Automated TopDraw and provides constrained based topology diagram. Uses H-bond from HERA, topology diagram from TOPs and PTGL	Sitavala A. *et.al* [4]	http://munk.csse.unimelb.edu.au/pro-origami/
10	Pro-Lego (2017)	Protein as Logo Blocks. Prolego analyze protein topology and propose a 1D contact string, from which 2D and 1D topology diagram has been	Khan *et.al.* [This paper]	www.proteinlego.com

		generated using developed JavaScript libraries.		

Above table describes a chronological study for methods developed till now for protein topology generation. For each method general working principle with corresponding reference and service availability has been provided in corresponding columns.

1.2 Contact string (CS) from Protein chain

Contract string is the linear construct of protein secondary structure (SS) adjacency matrix (Fig S2, lower panel). Along with SS contact information, this adjacency matrix contains type of contact and its orientation information. Detail contact definition has been listed in Table S2. In “contact string”, we decompose the adjacency matrix diagonally and construct a string, where each segment represents contact information of sequentially distanced neighbours in an increasing order. The segments are noted as dash “-” and each contact (element of adjacency matrix), as dots “.”. This construct of linear notation makes topology comparison, storage and visualization easy and efficient.

The definition of SS contact has been carefully estimated and selected [7]. The contact distance criteria is found to be in agreement to conventional use [8,9]. A brief description of building of adjacency matrix and “contact string” can be see in Figure S2.

A working example of contact string generation can be found in Fig. S2, for a 4 helix protein. Contact matrix(M) of (H x H) captures the contacting alpha helices as well as their orientation. Matrix dimension depends on the number of alpha helices (H) in the protein chain. The matrix element (*i,j*) shows the contact and orientation of two alpha helices i and j in the protein. If i and j are not contacting then M(*i,j*) is ’0’ ,otherwise can be ’a’,’r’ or ’p’ depending on helix orientation of anti-parallel, orthogonal and parallel respectively. The near diagonal elements represent contact between sequentially adjacent alpha helices (contact distance = 1) and as we go diagonally up the contact distance increases. Contact distance is the distance between two contacting alpha helices in terms of number of alpha helices they are apart in sequence. We have considered contact distance between same helices as ’0’, between adjacent as ’H’ as 1 and henceforth, represented by “-” in contact string.

Table S2 : SSE contact and orientation assignment

Type of Secondary Structure in-contact	Definition	Orientation
HH	Minimum of 3 residue contact	Anti-parallel (135<=Abs(θ) < 180)<br>Parallel (0<=Abs(θ) < 45)<br>Orthogonal (45<=Abs(θ) < 135)
EE	Minimum of 2 residues in contact	Anti-parallel (90<=Abs(θ) < 180)<br>Parallel (0<=Abs(θ) < 90)
HE/EH	Minimum of 3 residue in contact	Anti-parallel (135<=Abs(θ) < 180)<br>Parallel (0<=Abs(θ) < 45)<br>Orthogonal (45<=Abs(θ) < 135)

ProLego server application defines contact between two secondary structures (alpha (H), beta (E)), with above tabulated rules. The residue contact deinition has been adapted as per most popular definition of distance between heavy atom between a residue pair is sum of their VdW radius with a thresold of 0.6 Angstroms. The minimum number of “contacting residues” required for two SSE to be in contact has been described in the column “Definition”. Orientation defnitions of two contacting SSE has been followed as under the column “Orientation”.

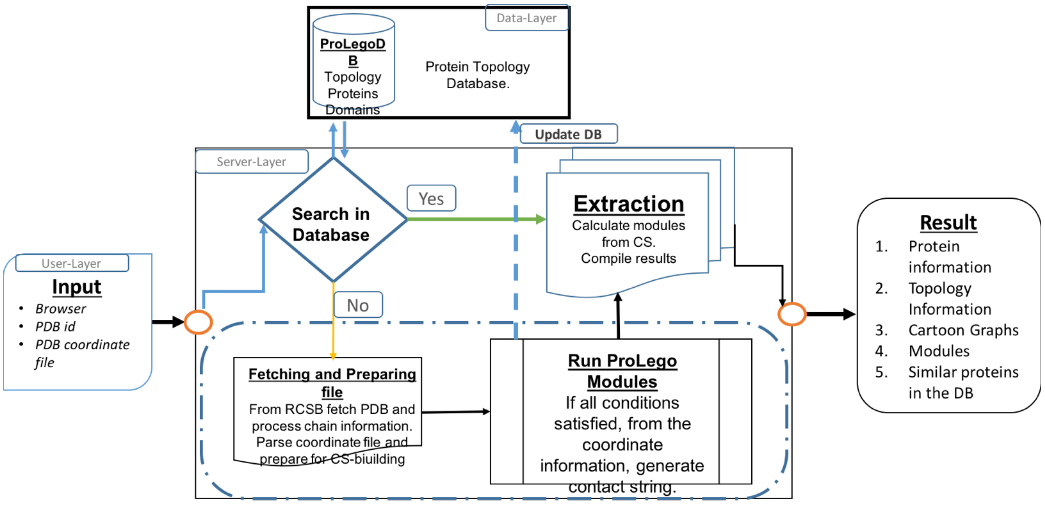

Figure S1 :ProLego architecture. The layered-architecture makes, ProLego easy to use and maintain. The user-layer has different option of input for protein chain. Layers are shown in different boxes. The pipeline shows the flow of instruction in black and blue lines.

1.3 Extracting topological modules using contact string

For different secondary structure content, presence of topological modules can be searched. Modules are the structural patterns of lower SS content occurring in higher SS content proteins. For example, a protein with 4 alpha-helix can have a structural pattern of two three alpha helix proteins. This can be generalized for "n" sse (∇ n> 4), where 3 to n-1 topological modules can be extracted . A pictorial representation of this concept has been described in Figure S2.

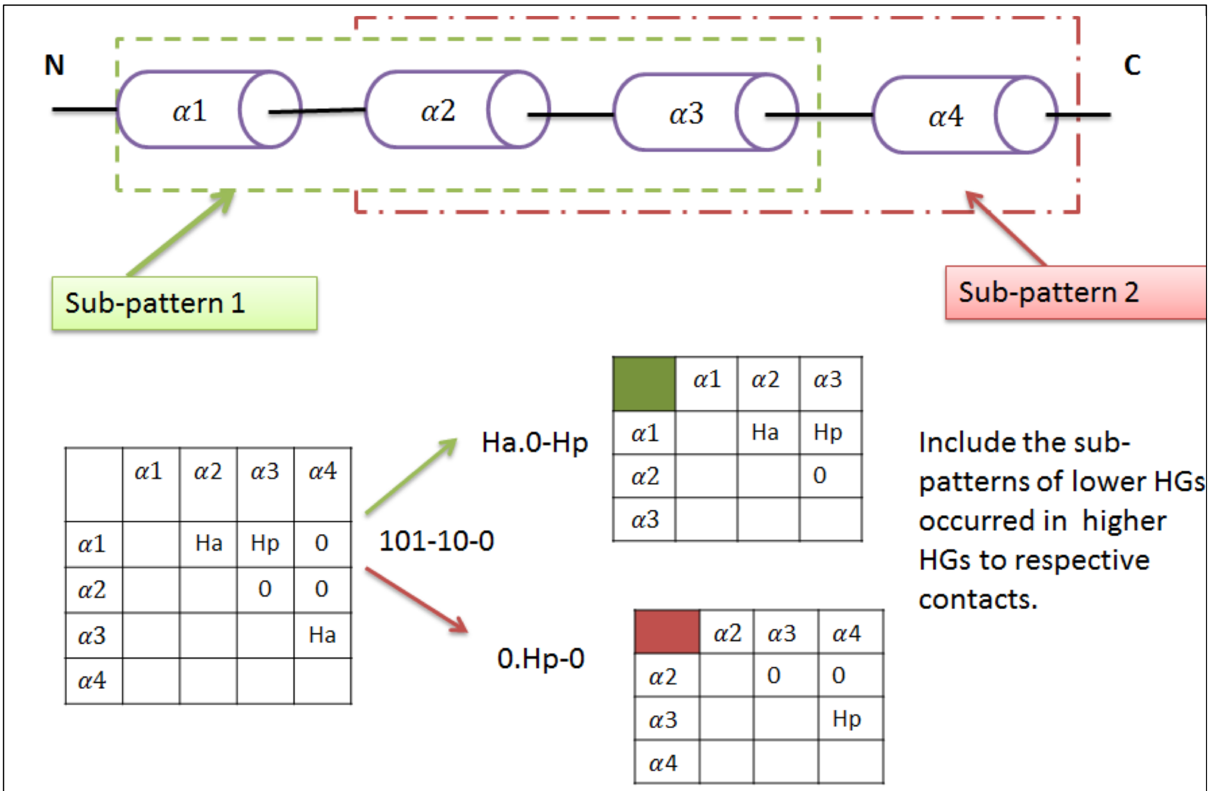

Figure S2: Construction of contact string and identifying sub-patterns using contact string. The adjacency matrix (bottom left), shown the contacts in a protein with 4-alpha helix. The diagonal decomposition of and construct of contact string can be seen. Analyzing protein chain from N to C terminal, two sub-patterns has been shown. Figure in the lower panel, shows contact matrix and contact string and corresponding sub-patterns.

This is also referred as “topological modules” that may be used to build and design more complex proteins with higher SS content from the structural roots of lower SS content proteins. Topological modules for different sets of SS has been formed from the contact string. A working example of chain L of photoreaction protein (PDB: 1JB0:L), has been provided in Table S3. This protein chain has 5 alpha helices. Using contact string, 8 different topological modules can be identified, which have significant presence in ProLegoDB.

With ProLego contact string, it is quite easy to identify and decompose repeating protein units. A example of Zink and Tax binding protein has been discussed in Figure S4. These proteins share sequence identity of ~19% but built with similar structural topology and topological modules. From fig. S4 A and B , the repeating unit of an antiparallel sheet and a helix can be observed with three different visualizers

Table S3: Extracting modules using topology string for Photoreaction centre protein 1JB0:L

#SSE	SSE string	Topology String	Protein DB	Domain DB
3	HHH	Hp.0-0	1NHL:A, 2ZI0:A, 3MP7:B, 3MQ7:A, 4N6J:A, 5B1A:I,	1lr1A00, 1y9bB02, 2r62A02,
3	HHH	0.Hp-0	2F60:K, 1K1F:A, 1OJH:A, 2P22:D, 2ZSI:B, 3CS5:A,3IV1:A, 4EFA:G, 4ICG:A, 5FIY:C, 5JIE:D,	1l1fA03, 1ojhC00,
3	HHH	Hp.Ha-Ha	1NH2:B, 1SKV:A, 2R9I:A, 2XQU:A, 3P8C:F, 3R84:A,3WKR:C, 3ZK1:A, 4MH6:A, 5D50:F,	1b06A01, 1bsmA01, 1idsA01, 1kkcA01, 1ma1A01, 1nh2B00,1p49A02, 1s5jA04, 1wb7A01, 3fx7A00, 3kkyA01, 3oaaG01,d1coja1, d1wb8a1, d2c2aa1,
3	HHH	Ha.Ha-Hp	2ERL:A, 2FCW:A, 2GOM:A, 2J5Y:A, 3MXZ:A, 3T47:A, 4FZP:A, 4NPD:A,5LB7:B, 1EZ3:A, 1GVN:A, 1LJ2:A,1QSD:A, 1WRD:A, 1Z8U:A, 2AHM:A, 2C5K:T, 2E2A:A,2FZT:A, 2V6Y:A, 2VQE:T, 2W2U:A, 2XF7:A, 2XVT:A,3A8Y:C, 3BBZ:A, 3EAB:A, 3GI7:A, 3GN4:A, 3L8R:A,3LOF:A, 3MQ1:A, 3MZW:B, 3PH0:C, 3RGU:A,3UUL:A, 3UUN:A, 4A5X:A, 4CQI:A, 4FM3:A, 4HKZ:H,4HWC:A, 4HWD:D, 4HWH:A, 4KBQ:D, 4M70:E, 4MBQ:E, 4NTW:C, 4PS2:A, 4TX5:A, 4U7I:A, 4U7Y:A, 4UI1:C, 4Z8L:B, 5FVK:A, 3A8Y:D,	1chuA03, 1dd5A01, 1eh1A01, 1ge9A01, 1gvnC00, 1h5wB01,1hx8A02, 1i6zA00, 1is1A01, 1iseA01, 1k04A02, 1knrA03,1m62A00, 1o3xA00, 1qsdA00, 1sumB01, 1uk5A00, 1uurA01,1wfdA00, 1wrdA00, 1x9bA00, 1yd8G00, 1z8uA00, 2c5kT00,2dl1A01, 2i0mA01, 2jwsA00, 2kdlA00, 2ptfB02, 2v6yA00,2v8sV00, 2vqeT00, 2w2uA00, 3a8yD00, 3axjA02, 3ldqB00,3mxzA00, 3nvoB02, 3qb5K02, d1chua1, d1cuna2, d1fpoa2,d1hcia4, d1lvfa_, d1qsda_, d1s35a1, d1u5pa1, d1u5pa2,d1uura1, d1vcta1, d1wrda1, d2ahma1, d2fcwa1, d2fzta1,d2uubt1, d3bvua1,
3	HHH	Ha.Hr-0	2CZS:A, 4YTW:A, 1JKO:C, 1VF6:A, 4HWF:A, 4JQU:B,4UZZ:B, 5FIR:H,	1dv0A00, 1jalA03, 1k6yA01, 1rsoB00, 1ug2A01, 1vf6A00,1vf6C00, 1whcA01, 1wvrA02, 1y74A00, 1y76A00, 2cqqA01,2dbyA03, 2elkA00, 2h5xA03, 2llkA01, 2m2eA00, 2ppyA02,2uzfA02, 2vx2G02, 2ztdA03, 3bvoB01, 3hrxA02, 3ihpB04,3l9dA02, 3qxiA02, 3rsiA02,
4	HHHH	0.Hp.Ha-0.Ha-0	5UNI:A,	
4	HHHH	Hp.Ha.Ha-Ha.Hp-Hp	3T49:A, 3H3M:A, 3N7S:C, 4XNG:D,	
4	HHHH	Ha.Ha.Hr-Hp.0-0		1avoB00, 1gsuA02,

Modules extracted by ProLego. From the topology string of 1JB0:L, constituent topological modules has been extracted and listed in above table. The table contains the topologies for which at-least one representative protein is found in ProLegoDB. Protein and domains are listed as their protein Id.

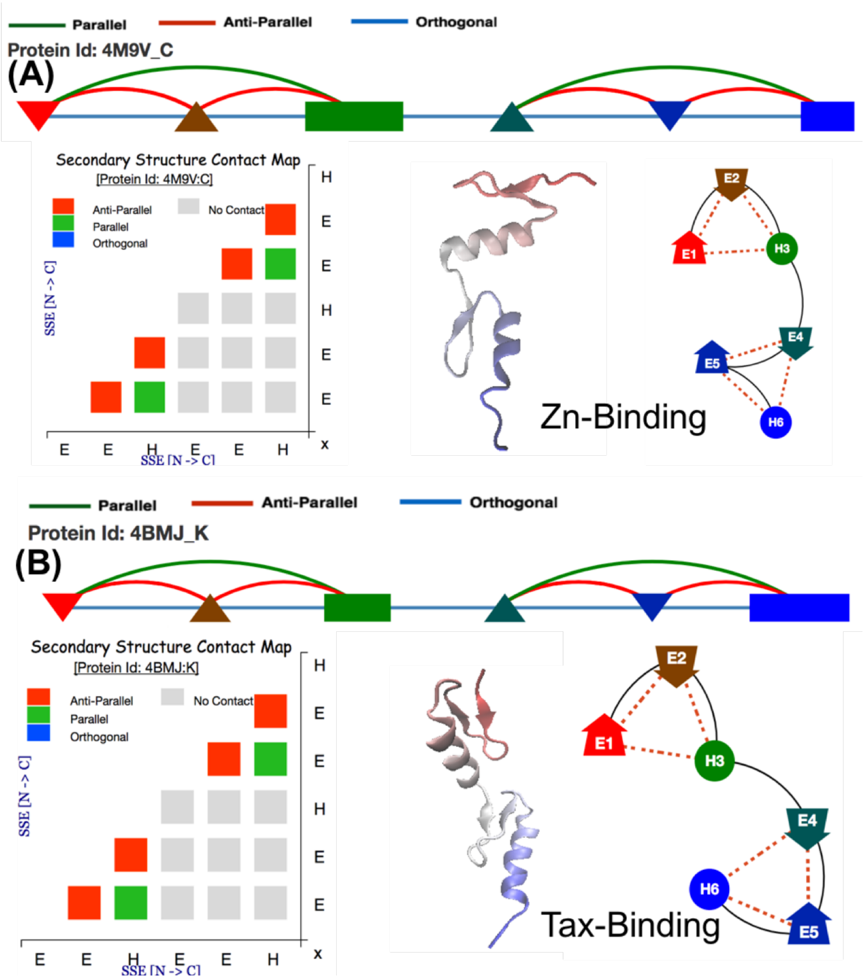

(C) Pair wise sequence comparison of 4M9V_C(ZN-binding) and 4BMJ_K (Tax Binding) protein chains

```
CLUSTAL O(1.2.4) multiple sequence alignment


ZN-binding      GPSERPFFCNFCGKTYRDASGLSRHRRAHLGYRPRSCPECGKCFRDQ---SQVNRHLKVH
Tax-binding     ----VHKKCPLCELMFPPNYDQSK-FEEHVESHWKVCPMCSEQFPPDYDQQVFERHVQTH
                        * :*   :    . *:  . *:  : : ** *.: *  :   . .:**::.*

ZN-binding      QNKP--
Tax-binding     FDQNVL
                 ::
```

<u>Percent Sequence identity: 19.64%</u>

Figure S3: Topology analysis of repetitive protein domains from Zinc-binding (PDBid: 4M9V_C) and Tax-binding proteins (4BMJ_K). Figure A and B, shows different representation of two proteins, which has a same topology but sharing very low sequence identity (19%, as shown in Fig C). For each case (A, B), upper diagram shows the linear topology with strands represented as triangles (with relative orientation as up/down triangle) and helices are represented as rectangle. The length of helical rectangles scaled as per number of residues in the helix. The protein chain is represented as red to green to blue as passes from N to C terminal. The linear lines, connecting secondary structure (SS) blocks shows the chain connectivity, whereas the arc lines represent the spatial connectivity and type of SS contact (color coded as labeled, Table S2). The secondary structure contact map, shows all spatial contact between pairs of SS. A 3D carton representation (VMD generated) and 2D topology cartoon plot as generated from ProLego. The 2D ProLego cartoon shows contact between two SS blocks by red dotted lines and chain connectivity by black continuous line.

Table S4: Color code representation of contact orientation between secondary structures in 1D topology diagram

Sl no	Color of Arc	Property : Orientation
1	Red	Anti-Parallel
2	Blue	Orthogonal
3	Green	Parallel

Above table lists the color definition used for arcs in linear or 1D topology graph.

Table S5 : P-value correction for topologically significant groups

SSE String[a]	Raw P-Value[b]	Rank[c]	BH-correction[d] (FDR < 0.001)
EEEE	3,66E-32	1	2,38E+06
EHEHEHEHH	2,57E-22	2	4,76E+05
EEEEEE	5,60E-08	3	7,14E+06
HHEEHEEEE	7,76E-05	4	9,52E+06
EHEEHE	1,76E-04	5	0.000119
EEHEEE	2,48E-03	6	0.000142
HHH	1,43E+00	7	0.000166
HHHHHHHH	4,94E-01	8	0.00019
EHEEHEH	9,76E+00	9	0.00021
EEEEEEEEEE	2,52E+01	10	0.000238
EEEEEEEEEEEEE	1,36E+01	11	0.000261
HHHHHHH	3,44E+02	12	0.00028
HHHHH	1,54E+03	13	0.000309523809524
HHHHHH	4,37E+03	14	0.000333
EHEHEHEHHEHEHHE	8,61E+04	15	0.00035
EEEHEEHE	1,46E+05	16	0.00038
HEEEEE	5,44E+05	17	0.00040
EEEEEEE	6,01E+05	18	0.0004285
EHHEHHEHHEHH	8,89E+05	19	0.000452
HEEE	1,12E+06	20	0.000476190
HEEH	1,36E+06	21	0.0005
HHHHHHEEEHHH	1,46E+06	22	0.000523809
HHHHHHHHH	1,48E+06	23	0.000547619
EHEHEE	2,09E+06	24	0.000571428

EEEEEEHHEEEEHHEE	2,61E+06	25	0.0005952
HHHEEH	2,77E+06	26	0.000619047
EHEEHEEE	2,99E+06	27	0.00064285
EHEHEHEHEHH	3,11E+06	28	0.0006666667
EEHEEHE	3,11E+06	29	0.00069047476
EEEHEHEE	3,68E+06	30	0.0007142857
EEHEE	7,23E+06	31	0.00073809
HHHHHHHHHH	0.00010751117673	32	0.000761904
EEEEEEEE	0.00011176222	33	0.00078571
HHHH	0.00012150197	34	0.000809
EEEH	0.00012345754	35	0.0008333
EEEEE	0.0001271066	36	0.00085714
HEEEEHHEEEEHHEEH	0.000203336	37	0.0008809
HEEHEEEE	0.0002697978	38	0.000904
EEEEEEEEE	0.000318217	39	0.00092
EEEEEEEEEEEE	0.000318217	40	0.000952
EEEEEEEEEEE	0.000656742	41	0.00097

The list of secondary structure groups ([a]) denoted by their SSE string, is the collection of proteins with same SSE composition from N to C terminal. The distribution of topologies in each SSE group are tested by Chi-Square test and sse-groups are picked as significant id the P-Value is less than 0.001. The raw P-value score for each group test has been listed under the column ([b]) Raw P-value. The multiple statistical test has been further examined by P-vlaue correction method to detect False discovery rate (FDR). Here we have shown the implementation of Benjamini-Hochberg [10]. *With false discovery rate less than 0.1%, the statistical significant values are reported.*

Table S6: Analysis of decoy sets (generated from I-TASSER) using ProLego (Decoy_filter module).

			P[1]	NP[2]	NC[3]	P[1]	NP[2]	NC[3]
1	1ten_	294	28	3	263	9.524	1.02	89.456
2	1o2fB_	510	96	66	348	18.824	12.941	68.235
3	2pcy_	435	13	34	388	2.989	7.816	89.195
4	1kjs_	548	10	12	526	1.825	2.19	95.985
5	1dtjA_	285	16	3	266	5.614	1.053	93.333
6	1mn8A	120	5	4	111	4.167	3.333	92.5
7	1gjxA	525	16	1	508	3.048	0.19	96.762
8	1ne3A	566	28	1	537	4.947	0.177	94.876
9	1tif_	542	6	3	533	1.107	0.554	98.339
10	1vcc_	551	5	1	545	0.907	0.181	98.911
11	1di2A_	374	1	1	372	0.267	0.267	99.465
12	1sro_	515	37	0	478	7.184	0	92.816
13	1mla_2	335	2	2	331	0.597	0.597	98.806
14	1thx_	302	3	4	295	0.993	1.325	97.682
15	1ogwA_	520	26	2	492	5	0.385	94.615
16	1bq9A	573	5	2	566	0.873	0.349	98.778
17	1orgA	442	0	1	441	0	0.226	99.774
18	1b72A	534	180	138	216	33.708	25.843	40.449
19	1abv_	526	6	3	517	1.141	0.57	98.289
20	1af7__	527	24	30	473	4.554	5.693	89.753
21	1g1cA	307	7	15	285	2.28	4.886	92.834
22	1bm8_	329	49	7	273	14.894	2.128	82.979
23	1tfi_	339	47	0	292	13.864	0	86.136
24	1aoy_	529	12	422	95	2.268	79.773	17.958
25	1r69_	291	8	4	279	2.749	1.375	95.876
26	1npsA	469	75	6	388	15.991	1.279	82.729
27	1itpA	526	62	16	448	11.787	3.042	85.171
28	1b4bA	460	200	16	244	43.478	3.478	53.043
29	1tig_	565	6	12	547	1.062	2.124	96.814
30	1gpt_	469	0	3	466	0	0.64	99.36
31	2f3nA	485	74	91	320	15.258	18.763	65.979
32	2a0b_	282	0	3	279	0	1.064	98.936
33	1gyvA	337	13	4	320	3.858	1.187	94.955
34	1csp_	315	16	4	295	5.079	1.27	93.651
35	1ah9_	510	15	1	494	2.941	0.196	96.863
36	2cr7A	540	50	73	417	9.259	13.519	77.222
37	1cqkA	284	5	1	278	1.761	0.352	97.887
38	1kviA	550	14	0	536	2.545	0	97.455
39	1fadA	514	0	1	513	0	0.195	99.805
40	1egxA	352	11	0	341	3.125	0	96.875
41	1dcjA_	525	15	2	508	2.857	0.381	96.762
42	1n0uA4	301	3	3	295	0.997	0.997	98.007
43	2reb_2	550	59	139	352	10.727	25.273	64
44	1jnuA	269	3	1	265	1.115	0.372	98.513
45	1fo5A	340	17	25	298	5	7.353	87.647
46	1pgx_	562	36	32	494	6.406	5.694	87.9
47	1gnuA	553	86	48	419	15.552	8.68	75.769

48	1mkyA3	285	10	6	269	3.509	2.105	94.386
49	1hbkA	300	11	9	280	3.667	3	93.333
50	1no5A	426	59	124	243	13.85	29.108	57.042
51	1shfA	536	34	0	502	6.343	0	93.657
52	256bA	506	397	38	71	78.458	7.51	14.032
53	1sfp_	308	15	14	279	4.87	4.545	90.584
Each row describes a set of protein with different number of decoys generated using I-TASSER. For each set, number of Non Contacting (NC) (1) topology in numbers and in percentage has been given. These are topologies which do not have any secondary structure contact. The rest topologies have been searched in ProLegoDb for their occurrence status and listed under prevalent (P) (2) and Non-prevalent (Np) (3) topologies.								

1.4 Decoy Filtering using ProLego Topology

Using ProLego, 53 non-redundant protein decoy sets has been analysed, to examine the contribution of different ProLego topology. The protein sets are considered from I-TASSER decoy set 2 and the raw decoys were generated from I-TASSER *ab initio* simulation. The decoys were structurally refined by GROMACS4.0 and OPLS_AA force field which has been taken from Zhang Lab as available with this address (zhanglab.ccmb.med.umich.edu/decoys/decoy2.html). The tested proteins have different composition of secondary structure (H/E numbers) and of varied size (range from 120 to 550 residues).

Using the standalone suit, contact string has been generated for decoys in each protein case. The *ab*-initio designing principle is filtered with energy contributions and a set of minimum energy templates has been listed as designed decoys. With ProLego topology we can scan the presence of different topology scaffolds, that depends on contacting secondary structures. While examining the decoys, we found that on an average ~85% (± ~15%) can be filtered out as they have "non-contacting (**NC**)" secondary structures. The rest "contacting" topology in ProLegoDB can be used to scan the "P" (prevailing) or "NP" (Non-Prevailing) topologies. As shown in Table S6, from all examined cases, on average ~8% of generated topologies can be mapped to "Prevalent" topology class. These selected topologies (~20 out of 200) can be used as top selected templates for further refinements.

Overall, ProLego can filter large number of decoys whose topologies are not found in the already studied non-redundant datasets. As for smaller proteins (with secondary structure number <= 10); the topology space has been exhaustively analysed and therefore the filter is

conventional. This data can be accessed from the github repository https://github.com/taushifkhan/plv-DecoySearch .

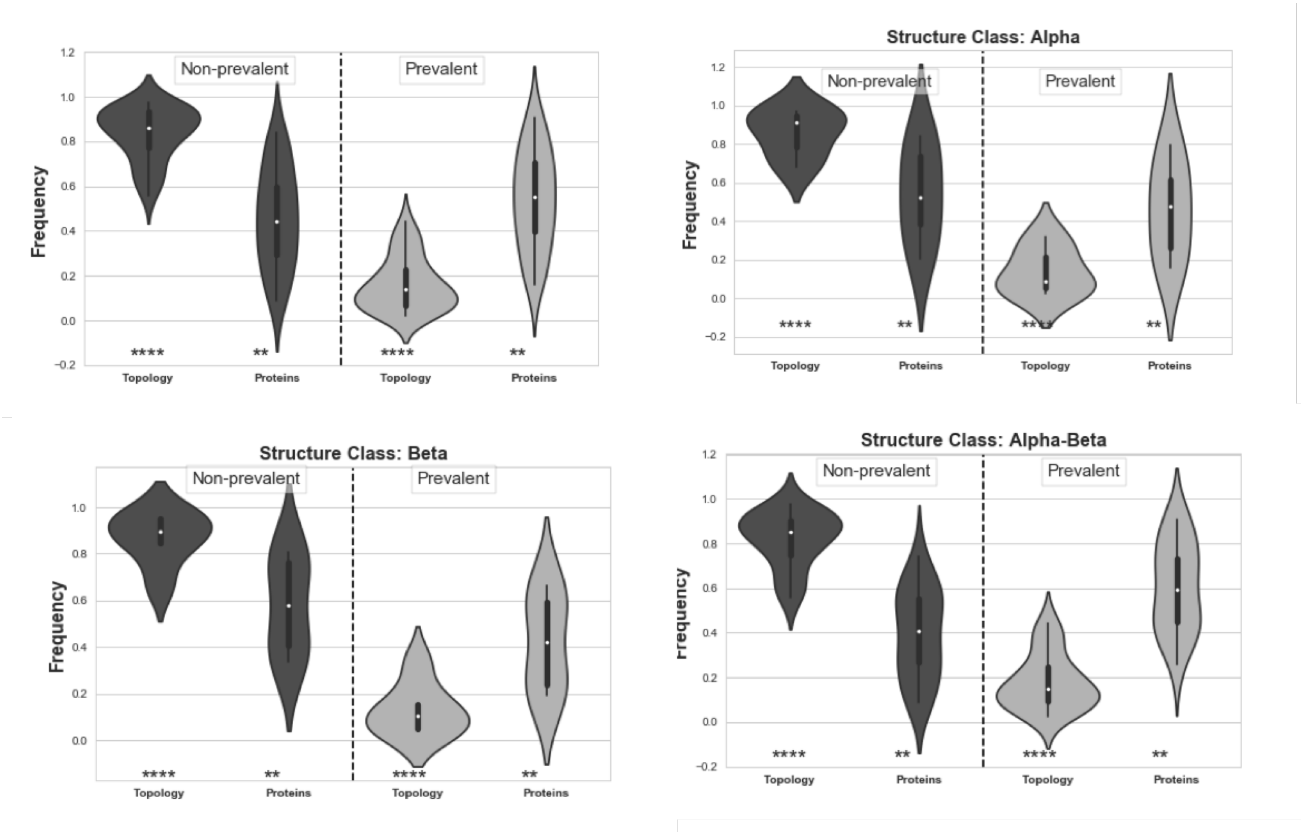

Figure S5: Structure class wise analysis of topology and protein distribution in groups of "Non-Prevalent" (left to dashed line) and "Prevalent" (right to dashed lines). Figure on the top left shows the data from all protein chains in ProLegoDB, where a sum of ~82000 protein chains and domains has been analyzed from non redundant datasets. These data has been categorized as per type of secondary structure e.g. Alpha (top right), Beta (left bottom) and Alpha-Beta (bottom right). The shape of violin plot describes the kernel density estimation of the distribution of data in different topologies and proteins. A summary of statistics can be drawn from the inner boxplot. The white dot represents the median, thick gray bar shows the interquartile range and thin line describes the 95% confidence interval. A clear distinction can be drawn on the nature of distribution of proteins as well as topologies in "Prevalent" and "Non-prevalent" groups. A comparison of distribution with non-parametric Wilcoxon rank-sum test has been performed and P-values are indicated as '' ('****':P-Val < 0.001 and '**' : P-val < 0.01) in the bottom*

1.5 ProLego in protein designing

Protein topology representation from ProLego is "string based". The inherent component based approach provides the relative ranking of different topology possible from a secondary structure (SS). We have compiled ProLegoDB, with topology information from the analysis of different non-redundant protein structure databases. At present, ProLegoDB has topology

information of ~7000 topologies, which can be mapped into different SS-groups (3 to 10 SS) and different sizes (residues 30 to 500).

Description of designed dataset:

Recently in the study and design of min-proteins (3 and 4 SS-group) , Rocklin *et.al*. reported important break though. In a systematic way, authors scans all possible topologies in 4 groups of SS-groups (HHH, EHEE, HEEH, EEHEE)[1] , with the objective to identifying the global determinant of folding and stability. The paper reported massive parallel testing of designed proteins based on Rosetta designing principles starting with 5000 to 40,000 designed proteins. Structurally unique set of designed main chain and near optimal sequence are then filtered for energy terms and 1000 designed templates are chosen, which are further assayed for sequence stability using chymotrypsin and trypsin. The feed-back designing principle has been carried out for 4 rounds and stability of 1000 designed main chains has been provided in the supplementary material of the paper (can be found in : http://science.sciencemag.org/content/357/6347/168/tab-figures-data). These synthetic designed proteins do not have any disulfides or metal coordination.

Experimental setup

From each design round, main chain topology has been extracted from the data provided in the above link using ProLego stand alone suit. Corresponding sequence stability score of the main chain as been categorized into stable (>1) and non-stable (<1) as reported by the authors. We have investigated the influence of topology classification ("prevalent" and "non-prevalent") in the reported designed topology pool. For each topology group (e.g. 'HHH'), occurrence of ProLego topologies and their stability has been monitored.

[1] Alpha Helix (α) : H; Beta Strands (β) : E. Protein chain is represented as string of secondary structure (H/E) from N to C terminal.

Results

Table S6: Designed proteins templates in ProLego topologies.

Topology HHH	ProLego Status	Round 1		Round 2		Round 3		Round 4	
		Stable	Not-Stable	Stable	Not-Stable	Stable	Not-Stable	Stable	Not-Stable
Ha.Ha-Hr	1	59	145	24	20	51	20	535	71
Hr.Ha-Hr	1	40	155	27	15	31	10	97	15
Ha.Hr-Hr	1	62	153	71	48	68	24	135	16
Ha.Ha-Hp	1	28	218	8	2	9	12	66	17
Ha.0-Hr	0	0	4						
Ha.Hr-Hp	0	2	7					1	0
Hr.Hr-Hp	1	9	56	7	11	8	3	26	5
Hr.Hr-Hr	1	5	21	3	2	9	2	8	2
Hr.Ha-Hp	0	0	1			1	2	3	0
Ha.Ha-0	1	0	3					1	2
Hr.Hr-Ha	0	0	3						
Hr.0-Hr	0	0	1						
Hr.0-Hp	0	0	1						
Hr.Ha-Ha	0	0	1						
Hr.Hr-0	1	0	1						
		**205**	**770**	**140**	**98**	**177**	**73**	**872**	**128**

ProLego topologies in 3H designed template (Data from Rocklin *et.al.* [11],). The rounds of experiments shows the enrichment in sequence stability. The stability score of >1 has been assigned as "stable" and <1 as "Not-stable". ProLego status of "1" and "0" represents "Prevalent" and "No-prevalent" topologies respectively.

The reported experiment has a feed-back design that enrich the sequence stability in each round. As shown in table S6, in subsequent rounds, number of stable designs is observed to be increasing (from ~20% in round one to 87% in round 4), which is according to the reported data. Here, we have investigated the population of stable and non-stable in each case of ProLego topology. As discussed in the main text, ProLego, examines all possible topology in a SS-group. In case of 3 helix SS, ProLegoDB has 50 different topologies and ~32% (16 out of 50) topology are "prevalent". Investigating the nature of topology space in the synthetic designing, we have observed near equal presence of "prevalent" (8) and "non-prevalent" (7)

topologies in round 1. Whereas, as the sequence enrichment occurred from round 2 onwards, there is a selective presence of "prevalent" topologies. Moreover, the number of "stable" folds in these topologies are found to be increasing in every "prevalent" topologies. We have seen similar observation of preferred topologies in other 3 cases (EHEE, HEEH, EEHEE), as shown in the datasheet.

This selective occurrence of certain topologies in the above synthetic dataset is in agreement with the "preferred" set in naturally observed topology dataset (reported in ProLegoDB). As shown in previous works [7], the "preferred" topologies can support functionally diverse scaffolds. This gives the evolutionary advantage for the frequent use of certain topologies in the structure space. As we have seen even in the de-novo synthetic datasets these naturally occurring topologies to be emerging as the "useful" scaffolds.

Table S7: Topology statistics

Case	**Total ProLego Topology**[1]	Round 1		Round 2		Round 3		Round 4	
Stable		**P**	**Np**	**P**	**Np**	**P**	**Np**	**P**	**Np**
HHH	15	99.024	0.976	100.0	0.0	100.0	0.0	99.885	0.115
EHEE	9	92.308	7.692	90.741	9.259	39.535	60.465	94.656	5.344
EEHEE	64	100.0	0.0	50.0	50.0	77.637	22.363	66.954	33.046
HEEH	85	0	0	5.0	95.0	13.333	86.667	0.0	100.0

Overall percentage occurrence of ProLego topologies in stable decoys of Rocklin *et. al.[11]*. For each SS constructs (case), different number of topology has been analyzed (1), which found to be present in ProLegoDB. All topologies are then grouped in to prevalent "P" and non-prevalent groups based on the described method. Above table, shows the percentage occurrences of prevalent and non –prevalent topology among "stable" decoy pool of each round.

1.6 ProLego time estimation

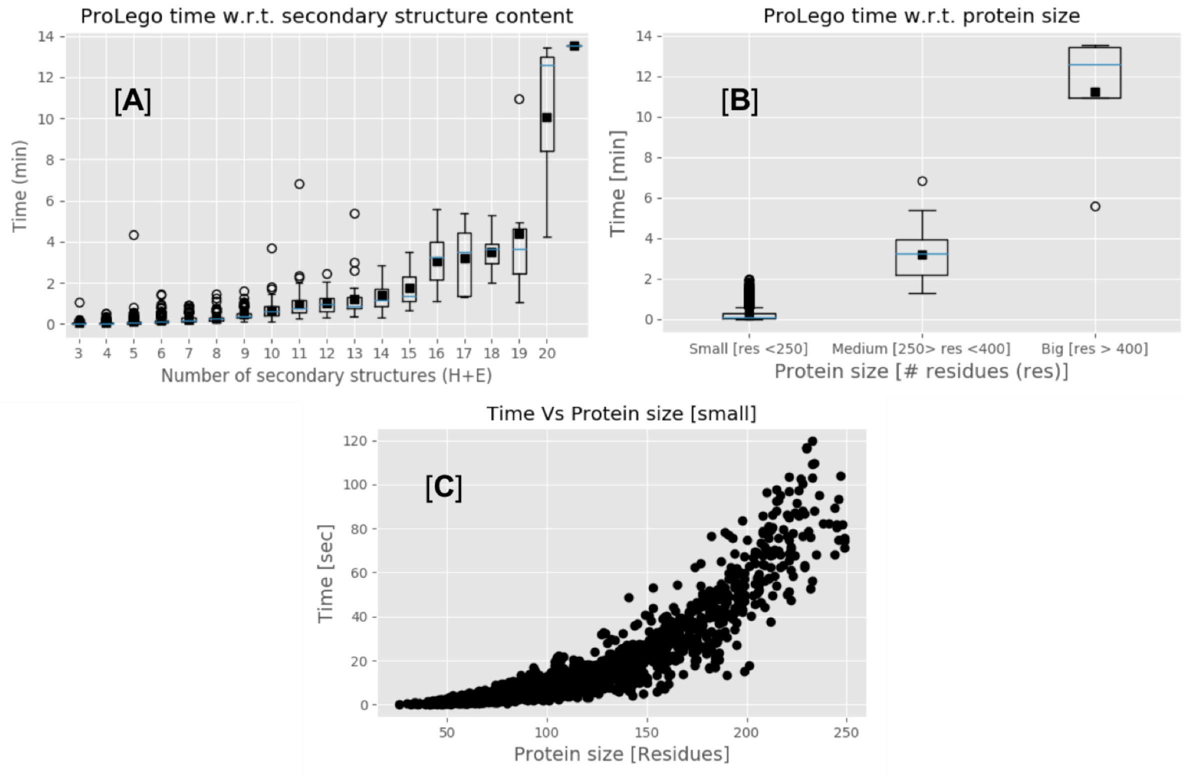

Figure S6: *ProLego time estimation from dataset of CATH S40 (v 4.1, 31170 proteins). Run time has been calculated for each protein and shown in above three panel. Figure A shows boxplots for time took for proteins for different total secondary structure content. Total secondary structure content is the sum of helices and strands in a protein. The mean of each case is shown as the square box. Figure B and C describes time with respect to residues as protein size. In Figure B, proteins have been grouped into three groups as 'small' (residues < 250), ''Medium' (residues between 250 and 400) and 'Big' (residues greater than 400), with similar properties as Fig A. For 'small' proteins, Fig C, shows time w.r.t total number of residues.*

ProLego calculation of contact string form proteins have observed steady increase with number protein size as shown by Figure S6. Analyzing protein size w.r.t total secondary structure content and total number of residues, time profiles are seems to be varying at different scales. For small proteins (i.e. total residues < 150), ProLego observed to be generating results with in 20 seconds, whereas when the number of residues went pass 200 total run time can be increased to 90 seconds. As shown in Fig S6(A), the mean value of total secondary structure content (< 10) is within 60 seconds.

In current implementation of ProLego, time estimation for small proteins are relatively fast, i.e. within 30 second. For bigger proteins, as the total secondary structure content increases (>

10 SS and total residues > 300), mean value of run time increase to 90 seconds with observable variation.

1.7 Detail of Dataset Analysis in ProLegoDB

Protein Data Base (PDB) has been filtered for X-ray structures with good resolution structures (< 3 Angstrom) and sequence identity clusters of 80%, 60% and 30%. The non-redundant subsets are generated from CD-Hit and PISCES server. The main goal of data variation is to check the consistency of the resulted topology groups and robustness of the prevalence classes.

Other Dataset tested:

- CATH (v. 4.1): Domains from Sequence cluster of 40.
- SCOP (v. 1.75): Domains from curated ASTRAL set of sequence identity 30 cluster.

In each dataset, protein chains have been analyzed in classes of SS-composition, which is defined by the arrangement of SS in protein chain from N to C terminal. In each composition group, protein chains are then clustered as per their topology. Statistical significance of each topology group has been computed by Chi-Square test. We consider the topology group distribution in a SS-composition is significant is the P-Value in < 0.001. i.e chances of finding such distribution by random is 1/1000.

The frequency of occurrence of topologies in a SS-composition ranked as per their percentile scores. The topology set which belongs to the first quartile (Q > 75) are grouped in "Prevalent (P)" topology group. The rest topologies are grouped as "Non-prevalent (NP)" topology group. We evaluate the statistical significance of difference between P and NP group using Wilcoxcon-Rank-Sum test. If the difference is found to be statistically significant (P-value < 0.01), we considered the subset of topologies as "prevalent" (or most-frequent).

Reference

1. Hutchinson EG, Thornton JM. A revised set of potentials for beta-turn formation in proteins. Protein Sci. [Internet]. 1994;3:2207–16.

2. Bond CS. TopDraw: a sketchpad for protein structure topology cartoons. Bioinformatics. 2003;19:311–2.

3. Veeramalai M, Ye Y, Godzik A. TOPS++FATCAT: fast flexible structural alignment using constraints derived from TOPS+ Strings Model. BMC Bioinformatics. BioMed Central; 2008;9:358.

4. Stivala A, Wybrow M, Wirth A, Whisstock JC, Stuckey PJ. Automatic generation of protein structure cartoons with Pro-origami. Bioinformatics. 2011;27:3315–6.

5. May P, Kreuchwig A, Steinke T, Koch I. PTGL: a database for secondary structure-based protein topologies. Nucleic Acids Research. 2010;38:D326–30.

6. Dalton JAR, Dalton J, Michalopoulos I, Michalopoulos I, Westhead DR. Calculation of helix packing angles in protein structures. Bioinformatics [Internet]. 2003;19:1298–9.

7. Khan T, Ghosh I. Modularity in protein structures: study on all-alpha proteins. Journal of Biomolecular Structure and Dynamics. 2015;33:2667–81.

8. Lesk AM, Kamat AP. Contact Patterns Between Helices and Strands of Sheet Define Protein Folding Patterns. 2007;876:869–76.

9. Konagurthu AS, Lesk AM. Cataloging topologies of protein folding patterns. J. Mol. Recognit. [Internet]. 2010;23:253–7. Available from: http://www.ncbi.nlm.nih.gov/pubmed/20151416

10. McDonald JH, University of Delaware. Handbook of Biological Statistics. 2009.

11. Rocklin GJ, Chidyausiku TM, Goreshnik I, Ford A, Houliston S, Lemak A, et al. Global analysis of protein folding using massively parallel design, synthesis, and testing. Science. 2017;357:168–75.
